# Climate Change and Mental Health: A Scoping Review

**DOI:** 10.3390/ijerph18094486

**Published:** 2021-04-23

**Authors:** Fiona Charlson, Suhailah Ali, Tarik Benmarhnia, Madeleine Pearl, Alessandro Massazza, Jura Augustinavicius, James G. Scott

**Affiliations:** 1 Queensland Centre for Mental Health Research, Queensland Health, Wacol, QLD 4076, Australia; suhailah.ali@uq.net.au (S.A.); m.gardner2@uq.edu.au (M.P.); James.Scott@qimrberghofer.edu.au (J.G.S.); 2 School of Public Health, The University of Queensland, Herston, QLD 4006, Australia; 3 Institute for Health Metrics and Evaluation, Department of Global Health, University of Washington, Seattle, WA 98195, USA; 4 Herbert Wertheim School of Public Health and Human Longevity Science & Scripps Institution of Oceanography, UC, San Diego, CA 92093, USA; tbenmarhnia@health.ucsd.edu; 5 Department of Health Services Research and Policy, London School of Hygiene and Tropical Medicine, London WC1E 7HT, UK; alessandro.massazza.13@alumni.ucl.ac.uk; 6 Department of Mental Health, Johns Hopkins Bloomberg School of Public Health, Baltimore, MD 21205, USA; jaugust6@jhu.edu; 7 Mental Health Programme, QIMR Berghofer Medical Research Institute, Herston, QLD 4076, Australia; 8 Metro North Mental Health Service, Herston, QLD 4006, Australia

**Keywords:** global health, climate, mental disorders, environmental health

## Abstract

Climate change is negatively impacting the mental health of populations. This scoping review aims to assess the available literature related to climate change and mental health across the World Health Organisation’s (WHO) five global research priorities for protecting human health from climate change. We conducted a scoping review to identify original research studies related to mental health and climate change using online academic databases. We assessed the quality of studies where appropriate assessment tools were available. We identified 120 original studies published between 2001 and 2020. Most studies were quantitative (*n* = 67), cross-sectional (*n* = 42), conducted in high-income countries (*n* = 87), and concerned with the first of the WHO global research priorities—assessing the mental health risks associated with climate change (*n* = 101). Several climate-related exposures, including heat, humidity, rainfall, drought, wildfires, and floods were associated with psychological distress, worsened mental health, and higher mortality among people with pre-existing mental health conditions, increased psychiatric hospitalisations, and heightened suicide rates. Few studies (*n* = 19) addressed the other four global research priorities of protecting health from climate change (effective interventions (*n* = 8); mitigation and adaptation (*n* = 7); improving decision-support (*n* = 3); and cost estimations (*n* = 1)). While climate change and mental health represents a rapidly growing area of research, it needs to accelerate and broaden in scope to respond with evidence-based mitigation and adaptation strategies.

## 1. Introduction

In 2009, a Lancet Commission on Climate Change asserted that “climate change is the biggest global health threat of the 21st century” [1]. In response, the ‘Lancet Countdown on health and climate change’ has been established as an independent, global monitoring system dedicated to tracking the health dimensions of the impacts of, and the response to, climate change [2]. While the Lancet Countdown includes numerous health indicators, it currently lacks an indicator capable of capturing the impact of climate change on mental health globally. This lack of representation in global climate and health initiatives is of considerable concern, as mental disorders are a leading cause of burden of disease globally and contribute to increased rates of premature mortality [3].

Climate change is expected to impact mental health via a range of direct and indirect pathways [4]. Direct pathways include exposure to traumatic events, such as bushfires and other severe weather-related events. Indirect pathways largely operate through a range of social, political, and economic determinants of mental health such as poverty, unemployment, and housing. Vulnerable people and places, especially in low-income countries, are anticipated to be particularly badly impacted [4].

Three recent reviews have attempted to synthesise the existing literature on climate change and mental health. Middleton et al., have explored the mental health impacts of climate change among Indigenous Peoples, populations in which climate change impacts are anticipated to be amplified [5]. Hayes et al. conducted a scoping review to explore an important question of how mental health might be integrated into climate change and health vulnerability assessments [6]. Cianconi et al. examined the association between climate change-related events and mental health; however, the review did not assess the quality of these studies [7]. Yet, assessing the quality of studies in such a novel and growing area of research is critical, as it helps identify research gaps and methodological issues that will help formulate recommendations for future studies.

The World Health Organization (WHO) has proposed five global research priorities for protecting human health from climate change: assessing the risks; identifying the most effective interventions; guiding health-promoting mitigation and adaptation decisions in other sectors; improving decision-support; and estimating the costs of protecting health from climate change [8]. Currently, there has only been the assessment of the evidence base across the first of these five research priority areas (assessing the risks).

Building upon existing reviews, this scoping review aims to assess the available literature and explore the key literature gaps, related to climate change and mental health across WHO’s five global research priorities for protecting human health from climate change. This is essential in order to understand the current gaps in the literature and inform future research priorities.

## 2. Methods

### 2.1. Scoping Review

This review followed the Preferred Reporting Items for Systematic Reviews and Meta-Analyses (PRISMA) 2020 guidelines [9] in conjunction with the PRISMA Extension for Scoping Reviews (PRISMA-ScR) [10]. To identify original research studies related to mental health and climate change, the following online databases were searched from 1 January 2001, to 31 December 2020: PubMed, PsycINFO, EMBASE, CINAHL, Web of Science, and Scopus. The PubMed search string (Box 1) was adapted for each database (search strings for each database can be found in the Appendix A). Whilst acknowledging there are other relevant and broader constructs of mental health, such as ‘emotional wellbeing’, we elected to constrain our search terms to draw a boundary around an otherwise unmanageable number of references. Additional studies were identified by examining the reference lists of key review articles. Three authors (F.C., S.A., and M.P.) independently conducted title/abstract and full-text screening using Endnote X9, and two authors (SA, M.P.) independently extracted data from studies that met the inclusion criteria into a Microsoft Excel sheet. Consensus was sought among these authors throughout this process.

The inclusion criteria were as follows:

- Must relate to mental health and climate change across one or more of the domains defined by the Lancet Countdown for Health and Climate Change 2018 [11]: climate change impacts, exposures, and vulnerability; adaptation, planning, and resilience for health; mitigation actions and health co-benefits; finance and economics; and public and political engagement
- Must be original research (e.g., cross-sectional or cohort studies)
- Publication year must be from 2001 onwards
- English language

Details of the protocol for this review were registered on PROSPERO (registration number CRD42020161076) and can be accessed at https://www.crd.york.ac.uk/prospero/display_record.php?ID=CRD42020161076.

Box 1. **PubMed search string.**

Search ((((((((((((“Climate Change”[Mesh]) OR “Global Warming”[Mesh]) OR “Global Warming”[tiab]) OR “climate change”[tiab]) OR “Greenhouse Effect”[Mesh]) OR “Greenhouse Effect”[tiab]) OR “Climatic Processes”[Mesh]) OR “Hot Temperature”[Mesh]) OR “Climate”[Mesh]) OR “Weather”[Mesh]) OR “Weather”[tiab])) AND (((((((“Mental Disorders”[Mesh]) OR “Mental Disorders”[tiab]) OR “Mental Disorder”[tiab]) OR “Mental illness”[tiab]) OR “Mental illnesses”[tiab]) OR “Mental Health”[Mesh]) OR “mental health”[tiab])

### 2.2. Quality Assessment

The same three authors (F.C., S.A., and M.P.) assessed all studies for which appropriate quality assessment tools were available (*n* = 93). We used the ‘Observational Cohort and Cross-Sectional Studies’ and ‘Case Series Studies’ Quality Assessment tools from the National Heart, Lung, and Blood Institute (NHLBI) [12]. The NHLBI tools were robustly developed and include individualised tools for a range of study designs (e.g., cohort, cross-sectional, case series). Qualitative studies were assessed using the Critical Appraisal Skills Programme (CASP) Qualitative Checklist [13]. Ecological studies were evaluated using a scale adapted from Cortes-Ramirez et al. [14], with the “sample size” and “validity of statistical inferences” criteria removed as their specificity was not deemed relevant to providing an overall quality assessment of the studies included in the review. We did not assess the quality of studies employing modelling, case report, time-series, case-crossover, panel, or other unique study designs due to the lack of appropriate quality assessment tools.

Studies were assessed as either ‘poor’, ‘fair, or ‘good’, guided by a series of questions set by the quality assessment tools. The findings of ‘poor’ quality studies were excluded from our synthesis of results but can be found listed in Table A1.

### 2.3. Research Framework

Studies were grouped by a member of the team under the five global research priorities for protecting health from climate change proposed by the World Health Organization: assessing the risks; identifying the most effective interventions; guiding health-promoting mitigation and adaptation decisions in other sectors; improving decision-support; and estimating the costs of protecting health from climate change [8]. Further information about the WHO framework can be found in Appendix A.

### 2.4. Figure Preparation

The PRISMA flow diagram was prepared using PRISMA2020: R package and ShinyApp for producing PRISMA 2020 compliant flow diagrams (Version 0.0.1) [15]. The world map was created using the *rworldmap* package (version 1.3-6) [16] in RStudio (version 1.3.1093) [17] with R 4.0.3 [18]. The graph of studies over time was also created in RStudio using *ggplot2* (version 3.3.3) [19].

## 3. Results

**Figure 1:**
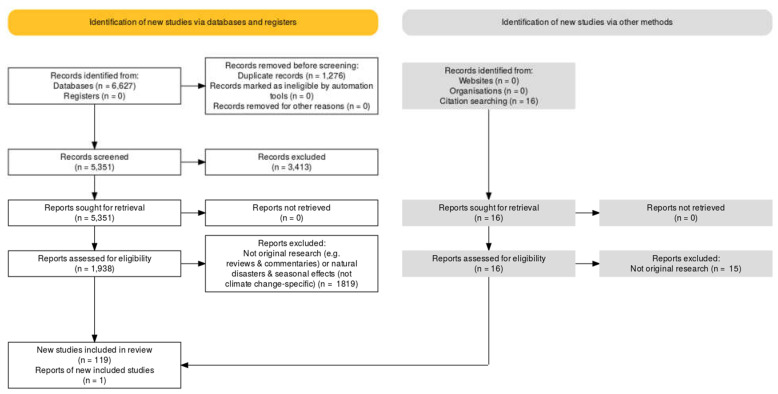
PRISMA flow diagram of study selection.

We identified 120 original research studies that examined mental health in the context of climate change-related exposures (Figure 1). Sixty-seven studies reported quantitative data, 34 reported qualitative data, and 19 reported a combination of qualitative and quantitative data. The most common study design was cross-sectional (*n* = 42), followed by case studies (*n* = 28), modelling studies (*n* = 12), ecological studies (*n* = 7) and cohort studies (*n* = 7). Table A1 provides a summary of the included studies.

**Figure 2:**
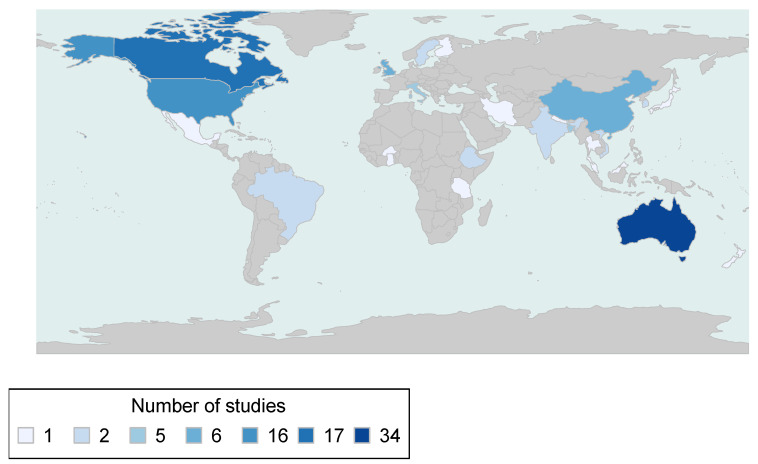
Map of included studies.

The geographies which represented the most original research studies were Australia (*n* = 34), Canada (*n* = 17), USA (*n* = 16), China (*n* = 6), United Kingdom (*n* = 6), Italy (*n* = 5), and Bangladesh (*n* = 5) (Figure 2). The majority of studies (77%) were carried out in high-income countries, with 12% from upper middle income, 12% from lower middle income and only 3% from low-income countries (as per World Bank classifications).

**Figure 3:**
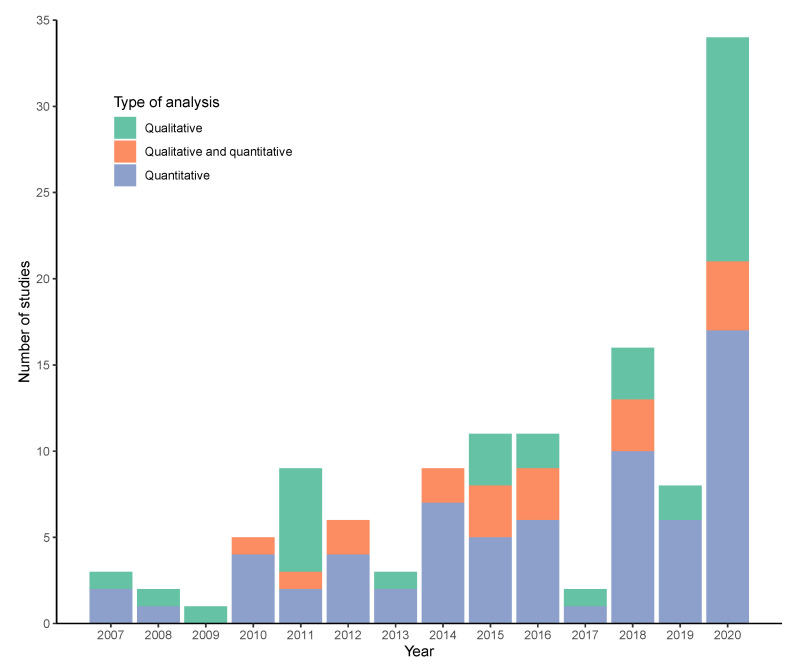
Number of studies published over time, by analysis type.

Although we searched for studies from 2001 onwards, we did not identify any published before 2007. There was an overall increasing trend in the number of studies between 2007 and 2020, with the highest number in 2020 (34 studies) (Figure 3).

The following sections provide a narrative summary of the original research corresponding to the WHO framework.

### 3.1. Assessing the Risks

The majority of studies (*n* = 101 out of 120) examined the mental health risks posed by climate change. The literature linked mental health outcomes to several climate-related exposures—heat, humidity, rainfall, drought, wildfires, and floods. Other environmental exposures, such as tropical cyclones and storms, were not included as no studies were found that explicitly referred to climate change. This section will present studies according to the following three categories (which are not mutually exclusive): (1) environmental exposures; (2) mental health outcomes; and (3) specific sub-groups and contexts that might be particularly vulnerable to the impact of climate change on mental health.

### 3.2. Environmental Exposures

#### 3.2.1. Temperature and Humidity

The most commonly examined environmental exposure in relation to health risk was temperature (*n* = 27), which was often measured alongside other meteorological variables such as humidity and rainfall. In most studies, an increase in temperature was positively correlated with poor mental health outcomes.

Three studies from Australia found associations between heat and mental health outcomes; however, the evidence was inconsistent. For example, Ding et al., report that an increase in temperature and humidity affects psychological distress, independently from pre-existing depression or anxiety [20]. Similarly, another study that looked at data from Adelaide, Australia, found that while there was a positive association between temperatures exceeding the maximum and minimum thresholds (32 °C and 16 °C) and psychiatric presentations to the emergency department, there was a significant decrease in this effect when temperatures were considered extreme [21]. A third study conducted on children in Australia showed a small effect of worsened childhood mental health with an increase in annual average daily maximum temperatures [22].

Several studies from Asia found that fluctuating temperatures influenced mental health and well-being, impacting productivity and livelihoods [23,24,25]. Long-term exposure to high and low temperatures in Taiwan resulted in a 7% increase of major depressive disorder incidence per 1 °C increment in regions with an average annual temperature above the median 23 °C. A higher incidence was found in the older age group (65+ years), with a slightly higher hazard ratio for males in this group, and females in the younger age group (20–64 years) [26].

Temperature also affected mental health and well-being in several studies conducted in North America. Noelke et al. reported that temperatures above daily averages reduced positive emotions like happiness, increased negative emotions like anger and stress, and increased fatigue [27]. Another study found that monthly temperatures above 30 °C increased the probability of mental health difficulties by 0.5%, and ongoing exposure to increasing temperatures (1 °C over five years) was associated with a 2% increase in the prevalence of mental health issues [28]. High ambient temperatures were also found to increase help-seeking for mental health, such as crisis text line usage in young adults in several urban areas in the United States [29]. A study exploring protective factors in the context of extreme seasonal weather in Tennessee, USA found that social cohesion was associated with reduced mental health impacts among low- and moderate-income residents [30].

#### 3.2.2. Drought and Rainfall

Drought was another frequently studied climate exposure (*n* = 16). All but one of the studies were from Australia and primarily focussed on rural communities. Almost half of these studies were based on large, longitudinal studies and utilised standard mental health measures such as the Kessler Psychological Distress Scale (K10). A nationally representative cross-sectional survey of Australian residents found that while drought was associated with elevated psychological distress (as measured by the K10) in rural communities, this was not the case for urban dwellers [31].

A longitudinal cohort study showed elevated drought-related stress and psychological distress (as measured by the K10) among farmers [32]. Drought-related stress, which captures worry about the impacts of drought on themselves and their families and communities, was influenced by socio-demographic and community factors (e.g., loss, government compliance pressures, and difficulties accessing mental health support or receiving inappropriate mental health services) that differed from the factors that influenced psychological distress [33]. Factors associated with higher psychological distress included being unemployed and exposure to other adverse life events, while protective factors included financial security, social support, and a sense of community [32,34]. The evidence for differential impacts on gender and age is conflicting [35,36,37].

One multi-country study has looked at rainfall as a climate-related exposure and has reported that the highest prevalence of mood disorders is observed in countries characterised by small variations across monthly rainfall and high levels of rainfall [38].

#### 3.2.3. Wildfire

Two studies from North America examined the impacts of wildfires on mental health. Qualitative interviews of people purposively sampled to include a broad cross-section of backgrounds and experiences from a severe 2014 wildfire season in the North-West Territories of Canada, in the context of climate change, reported how experiences of evacuation and isolation, as well as feelings of fear, stress, and uncertainty, contributed to acute and long-term negative impacts on mental and emotional well-being [39]. Prolonged smoke events were linked to respiratory problems, extended time indoors, and disruptions to livelihood and land-based activities, which negatively affected mental well-being [39].

A household study conducted one year after the Wallow Fire in Arizona, United States, concluded higher solastalgia, a term used to indicate distress caused by environmental change (measured using a scale adapted from [40]), and an adverse financial impact of the fire was associated with clinically significant psychological distress (as measured by the K10). In contrast, a higher family functioning score was associated with less psychological distress [41]. Other countries that experience regular wildfires, such as Australia, referred to as bushfires, had no research exploring mental health impacts in the context of climate change.

#### 3.2.4. Flood

Several studies examined the mental health impacts of flooding. An Australian study qualitatively explored individuals’ experiences from rural communities, concluding that the threat of drought and flood are intertwined and contributed to decreased well-being from stress, anxiety, loss, and fear [42]. A cohort study from the UK looking at the long-term impact of flooding found psychological morbidity persisted for at least three years after the flooding event [43].

In addition to the tangible mental health impacts related to exposure to specific climate change-related events, studies have reported psychological impacts of perceived risks associated with climate change [44,45].

### 3.3. Mental Health Outcomes

#### 3.3.1. Symptom Scales and Screening Tools

Symptom scales were commonly used to examine mental health outcomes. Distress (*n* = 17) was one of the most investigated outcomes; primarily psychological distress measured using the K10. Some studies attempted to develop specific environmental distress measures, including a scale for solastalgia and a specific ‘climate change distress’ scale [41,46,47,48]. Four studies used validated screening tools for mental disorders [39,41,45,46]. Only one study used a structured diagnostic instrument to determine mental disorder diagnoses [49].

#### 3.3.2. Hospital Admissions

Alternatively, many studies utilised routinely collected administrative data. Psychiatric hospital admissions associated with temperature and heat waves were frequently studied (*n* = 15). These studies demonstrate a positive association, particularly with elevated temperatures, with susceptibility varying in terms of demographic variables (e.g., older people shown to be at higher risk) [50,51] and type of disorder (e.g., organic disorders such as dementia) [52]. The relationship between hospital admissions and heat has been examined for a range of mental and neurological disorders, including dementia, mood disorders, anxiety disorders, schizophrenia, bipolar disorder, somatoform disorders, and disorders of psychological development [52,53,54].

#### 3.3.3. Mortality

Mortality has also been found to be influenced by high ambient temperatures for people living with mental illness and neurological conditions. A study of health outcome data from Adelaide, South Australia, for 1993–2006, has demonstrated that mortality attributed to mental and behavioural disorders increased during heat waves in the 65- to 74-years age group and in persons with psychosis [52,55]. Another European study supports this finding with increased mortality risk for people with psychiatric disorders during heat waves from 2000 to 2008 in Rome and Stockholm, particularly for older people (75+) and women. A study from England found that primary care patients with psychosis, dementia, and alcohol or substance misuse experienced significantly higher heat-related mortality above regionally defined temperature thresholds. This effect was more marked for younger patients and those with a primary diagnosis of alcohol or substance misuse [56,57]. Nitschke et al., found a small increase in mental health-related mortality in people aged 65–74 years during heat waves in metropolitan Adelaide [58]. Projections of mortality under different climate change scenarios in China also estimate increasing trends in heat-related excess mortality for mental disorders but a decreasing trend in cold-related excess mortality [59].

#### 3.3.4. Self-Harm

Temperature has also been associated with self-harm and suicide rates [60,61,62,63,64]. Data from Finland found that temperature variability explained more than 60% of the total suicide variance over several decades [62]. Using data from the US and Mexico, suicide rates were found to increase by 0.7% and 3.1%, respectively, for a 1 °C increase in monthly average temperature, with unmitigated climate change projected to result in a combined 21,770 (95% CI 8950–39,260) additional suicides by 2050 [64].

#### 3.3.5. Burden of Disease

Studies attempting to quantify the burden of mental disorders attributable to climate change are sparse. One study from South Korea has estimated the burden of disease related to climate change for a range of conditions and climate-related measures, including PTSD, as an outcome of disasters [65]. Unfortunately, this study was limited in several ways, including a lack of availability of input data.

### 3.4. Vulnerable Populations and Life Stages

#### 3.4.1. Pre-existing Mental Illness

We identified a body of research related to populations and contexts that are anticipated to be more vulnerable to climate change and its mental health impacts. People taking certain psychotropic medications (including hypnotics, anxiolytics, and antipsychotics) are at increased risk of heatstroke and death as a result of high temperatures, possibly due to disruptions in thermoregulation triggered by some psychotropic medications [56,66]. One study from Australia showed that people living with obsessive-compulsive disorder (OCD) experience obsessions and compulsions directly aligned with climate change concerns [67].

#### 3.4.2. Youth

According to the results of a national survey of Australian children aged 6–11, higher temperatures may impact children’s mental health [22]. Youth affected by drought may also experience cumulative mental health impacts [68].

#### 3.4.3. Indigenous People

Qualitative studies reporting the unique mental health impacts of climate change on Inuit communities in Canada have described a loss of place-based solace, land-based activities such as hunting, and cultural identity due to changing weather and local landscapes [69,70]. Changes in sea ice stability and surface area are of particular cultural concern for Inuit [71]. Studies among these communities report that climate and environmental changes increase family stress, enhance the possibility of increased drug and alcohol use, amplify previous traumas and mental health stressors, and may increase the risk of suicidal ideation [72]. Indigenous communities from Alaska have also expressed significant concern for climate change and its impacts on local sustenance resources, such as berries [73]. Mental health, physical health, traditional/western education, access to country food and store-bought foods, access to financial resources, social networks, and connection to Inuit identity emerged as key components of Inuit adaptive capacity in the face of climate change [74]. Studies conducted with Aboriginal and Torres Strait Islander peoples from Australia also highlight the environmental impacts of climate change on emotional wellbeing, including increased community distress from deteriorating the connection to country [75,76]. Heat also appeared to be associated with suicide incidence in Australia’s Indigenous populations; however, other socio-demographic factors may play a more critical role than meteorological factors [77].

#### 3.4.4. Low- and Middle-Income Countries

A study from Ethiopia, a low-income country that has a high dependency on the local environment to meet basic human and animal needs, demonstrated that seasonal environmental changes related to water security expose populations to significant emotional distress [78]. Small island developing states in the Pacific Ocean have been deemed to be particularly at risk and vulnerable to the impacts of climate change. Low-lying villages in the Solomon Islands in the South Pacific report that sea-level rise causes fear and worry on a personal and community level [79]. Research from Tuvalu, another small Pacific Island experiencing the threat of sea-level rise, describes the individual experiences of distress in the face of climate change and underscores the necessity to provide access to culturally informed social and mental health services in the region [80]. Three studies from Bangladesh have reported negative effects on emotional well-being as a result of climate-induced immobility [81,82,83].

### 3.5. Identifying the Most Effective Interventions

There were eight studies related to interventions; however, these were primarily exploratory and qualitative and focused on health professionals and community settings, and did not provide clear support for any one specific intervention.

One study looked at the health and social service responses to the long-term mental health impacts of a flood event. It concluded that sustained recovery interventions rooted in local knowledge and interdisciplinary action were required and that there are unintended consequences related to psychosocial interventions that can incite complex emotions and impact psychosocial recovery [84].

A study from rural Australia has described how a community development model, incorporating elements of health promotion, education, and early intervention, was accepted and considered effective in helping communities build capacity and resilience in the face of chronic drought-related hardship [85]. Unfortunately, this study did not measure the intervention’s impact on the mental health status of participants. Another Australian study that focused on rural health service managers found that 90% of respondents perceived climate change as likely to impact mental health and highlighted the important role of rural health services in education and advocacy on climate change’s health impacts [86]. A case study of health promotion practices that address climate change in Victorian (Australia) healthcare settings found that community gardens improved social connectedness and mental and physical health [87]. Results of a study from Canada revealed the need for improved medical education on climate change and health, including mental health, suggesting that a 3-h education activity would be useful and would allow family physicians to use this knowledge in their daily practice, notably through prevention and counselling [88].

Two studies explored the relationship between pro-environmental behaviour and mental health in terms of awareness of health risks and potential health co-benefits. Results from a survey in China indicated that residents’ health-risk perception is positively affected by climate-change information. Additionally, perceiving climate change as detrimental to mental health was shown to influence a residents’ attitude and intention to take environmental action more than physical health-risk perception [89]. A study of UK households found that while individual-level pro-environmental attitudes were negatively associated with mental health, household pro-environmental behaviours and attitudes of other household members were associated with positive outcomes, pointing towards a role of social capital and the household level as an important target for interventions [90].

Finally, a study exploring health perceptions in climate-driven migrants found that emphasis was placed on the importance of mental health, indicating that mental health interventions could be effective in this population [91].

### 3.6. Guiding Health-Promoting Mitigation and Adaptation Decisions in Other Sectors

A small number of studies (*n* = 7) examined how sectors outside of health can mitigate and adapt to reduce climate change impacts on mental health. Three of these studies were from Inuit communities. Social and environmental factors that may protect mental health and wellbeing were highlighted, including being on the land and connecting to Inuit culture, as well as how Inuit-led monitoring of environmental conditions can guide climate change adaptation that considers intangible losses and damages to well-being and ways of living [92,93,94]. A study from Nepal revealed similar themes [95], while a 15-year prospective cohort study of Hurricane Katrina survivors (New Orleans, LA, USA) has concluded that mitigation efforts should prevent trauma exposure through investments in climate resilience and eliminating evacuation impediments [96].

Barriers to implementing strategies to adapt to combat the agricultural impacts of climate change have resulted in negative mental health outcomes among subsistence farmers in Burkina Faso [97]. Barriers comprised financial and time constraints, material and labour shortages, and inaccessible information; however, awareness of climate change was also reported to be limited.

Finally, an exploratory study surveying people affected by widespread public safety power shutoffs (PSPS) to reduce the risk of wildfires in California found that while people were mostly supportive of PSPS as an adaptation measure, it was associated with poorer physical and mental health [98].

### 3.7. Improving Decision-Support

Very few studies (*n* = 3) reported issues that could support improved decision-making around mental health and climate change. Between 2010 and 2012, the WHO Division of Pacific Technical Support led a regional climate change and health vulnerability assessment and adaptation planning project in collaboration with health sector partners in 13 Pacific Island countries. Mental health was identified as a high-priority climate-sensitive health risk in eight out of the 13 countries [99]. However, another study found that policy documents on climate change from 12 countries made little or no reference to nine vulnerable groups, including people with mental illness [100].

A study designed to develop and validate indices of adaptation to flooding found that people who perceived a risk of flooding in their home in the next five years adopted more preventative behaviours and adaptation behaviours than those who perceived little or no risk at all. Additionally, people who felt more adverse effects of flooding on their physical or mental health tended to adopt more adaptive behaviours than those who felt little or no adverse effects on their health [101].

### 3.8. Estimating the Costs of Protecting Health from Climate Change

We did not find any studies that assessed the costs associated with climate change impacts on mental health. However, one study from the United Kingdom found that a social prescribing service for mental health—a service model which supports people to access healthcare resources and psychosocial support, considered to hold potential for reduced cost and carbon-footprint—was associated with reduced financial costs but an increased carbon footprint per patient. However, none of the differences between groups reached statistical significance [102].

### 3.9. Quality of Studies

Overall, the studies identified in this review were of sound (fair to good) quality. Out of the 93 studies that were quality assessed, 75% were rated good, 23% fair and 2% poor. It is important to note that we found significant variability in how environmental exposures were measured. Among the quantitative studies, we identified two types of approaches to measure exposure to climate-sensitive environmental hazards: (i) self-reported exposures using questionnaires and interviews (note that all qualitative studies used self-reported exposures) and (ii) interpolated exposures using external sources of data.

Fifty-two studies relied on self-reported information to assign a climate change-related exposure to study participants. We found large variability across the survey questions used to capture self-reported exposures, as no standardised questionnaire exists (in contrast to self-reported mental health outcomes) to measure subjective experience regarding climate-sensitive environmental hazards. Specifically, each study in our selected sample used a different question or metric to capture the environmental exposure. Such variability makes the evaluation of the robustness of used tools and the comparison between studies difficult. To the best of our knowledge, we did not identify any self-reported tool regarding the exposures of interest that were validated beforehand.

In addition, 48 studies relied on interpolated exposures using various types of available meteorological data sources. Some studies used meteorological data (mostly precipitation and temperature) from monitoring stations in the area of interest, often retrieved from the national or regional bureau of meteorology or data service centres. Other studies relied on remote sensing data from various satellite products with reanalysis, including the PRISM Climate Group [103] or the Berkeley Earth Surface Temperature Dataset [104]. While some studies used the meteorological variable directly in their analysis, such as daily maximum temperature, others further calculated indices or metrics to define weather events such as drought and heat waves. Most studies used the finest spatial resolution that was available while some used large-scale measurements when assigning exposure to meteorological variables, which may lead to important misclassification biases and impact effect estimates of interest. Indeed, assigning the same exposure to temperature, for example, on a given day to all individuals in a country or region is unlikely to accurately represent individuals’ exposure given well-documented local variations in microclimates [105,106]. We also found differences in the temporal scales that were used across studies. While most studies relied on daily estimates to capture acute effects on mental health outcomes, other studies used monthly or annual estimates to capture chronic impacts, describing various mechanisms such as economic insecurity or migration. Finally, some studies provided limited details regarding data sources and how indices were calculated, making it difficult to understand how exposures were estimated and hindering reproducibility efforts.

## 4. Discussion

This scoping review is the first to explore the existing original research literature that investigates climate change and mental health using WHO’s global research priorities as a framework [8]. We identified 120 original studies published between 2001 and 2020 that specifically referenced climate change impacts, adaptation, mitigation, and other interventions relevant to mental health. Most studies were quantitative, used a cross-sectional design, were conducted in high-income countries, and were concerned with assessing the mental health risks associated with climate change-related exposures.

The literature consistently points to the negative associations that climate change-related events have with individuals’ and communities’ mental health. Climate change-related events were shown to be associated with psychological distress, worsened mental health (particularly among people with pre-existing mental health conditions), increased psychiatric hospitalisations, higher mortality among people with mental illness, and heightened suicide rates. These results are largely in line with the detrimental impacts that climate change has been shown to have on many other aspects of health [2].

While the overwhelming focus on the mental health risks of climate change is understandable and has generated insight and traction for advocating the importance of considering mental health within climate change, only four studies have focused on protective factors or the coping mechanisms people and communities have used when responding to the detrimental mental health impacts of climate change [26,38,90,105]. An in-depth understanding of which factors constitute ‘resilience’ in the face of climate change would contribute to a more nuanced picture of the associations between climate change and mental health [107] and could be useful when devising programs and policies for those most heavily impacted by climate change.

Additionally, the focus on characterising and quantifying the linkages between climate change and mental health has not been matched by applied research on addressing and reducing these associated mental health risks [8]. Future work should investigate more systematically the possible effectiveness, scalability, and cost-effectiveness of interventions and policies aimed at safeguarding mental health in the face of climate change, including non-mental health interventions. Various health professionals, including nurses [108], emergency department clinicians [109,110], clinical psychologists, and psychiatrists [111,112], have already outlined ways to address mental health problems related to climate change within clinical practice and broader policy, highlighting the strong willingness to act in the medical community. There is also a growing movement of climate advocacy in the health sector, recognising the responsibility to protect the health of current and future generations and calling for meaningful government action [113].

The current review points to some important avenues for future research. As mentioned above, one key research priority is to dissect the association between mental health and climate change by investigating possible mediators and moderators as well as risk, vulnerability, and resilience factors in different populations. A systems-thinking approach, as advocated by Berry et al., might be particularly suitable for this endeavour [114]. This will be aided by more precise operationalization and conceptualization of environmental and mental health variables in the climate change space, another important research priority. Recent work to devise precise measurement instruments for constructs such as climate anxiety [115] represents an important step in this direction. Additionally, in order to move beyond the assessment of risk, other important research priorities relate to understanding how climate change adaptation and mitigation strategies might impact mental health and wellbeing [98] and how engagement in different types of pro-environmental behaviours such as climate activism might impact mental health [116]. A more comprehensive discussion of future directions in climate change and mental health research and practice will be covered in a separate paper by the authors.

The current review has a number of implications for policymakers and intergovernmental organizations such as the World Health Organization. While WHO is increasingly directing its attention to the health impacts of climate change, mental health has generally remained absent from these conversations. For example, as part of the Health and Climate Change Country Profile Project WHO 2019–2020 cycle, WHO reported that only three (i.e., Antigua and Barbuda, United Arab Emirates, and Solomon Islands) out of the 10 recently reviewed countries included mental health and well-being outcomes as part of their climate-sensitive health outcomes monitoring systems [117]. This is in stark contrast with other conditions such as vector-borne and waterborne disease, for which monitoring systems were in place for nine out of the 10 recently reviewed countries. The current review highlights the need for monitoring systems to systematically include mental health as part of their indicators in order to ensure precise monitoring and availability of data at the national level. Additionally, it indicates the importance of including climate change as an important determinant of mental health in policy documentation and action plans, such as in the future extension of the WHO Mental Health Action Plan 2013–2020 [118] to 2030. Importantly, future work might want to explore whether existing WHO documentation and intervention plans for humanitarian settings such as the mhGAP-HIG [119], the Building Back Better framework [120], and scalable psychological interventions [121] are applicable in the context of climate change or whether more specific adaptations are needed. Finally, WHO’s recent work on the integration of mental health and psychosocial support (MHPSS) within disaster risk reduction efforts [122] might represent an important opportunity to integrate climate change within MHPSS.

Palinkas et al. have reviewed the preparedness and response strategies from a mental health services perspective across three areas: (1) acute and extreme weather events; (2) sub-acute or long-term events; and (3) the prospect of long-term and permanent changes, such as an uninhabitable physical environment [122,123]. It is these long-term impacts, in particular, which may demand a shift in attention, as we found only one study which examined the mental health impacts of more general and permanent environmental degradation [124].

A substantial limitation in the surveyed literature concerns the under-representation of research from low- and middle-income countries. The majority of studies identified in this review were conducted in Australia, Canada, and the US. However, evidence indicates that low- and middle-income countries are more at risk of climate change-related events and have fewer resources to respond to these stressors due to pre-existing vulnerabilities [125,126]. For example, according to the Climate Change Vulnerability Index, the five most vulnerable countries in the face of climate change globally in 2016 were Central African Republic, Democratic Republic of Congo, Haiti, Liberia, and South Sudan, all low-income countries considered at “extreme risk” in the face of climate change [127]. These vulnerable regions and populations are among the least responsible for the greenhouse gases that are warming the planet. The concept of climate justice highlights this double inequality of climate change, where there is an inverse distribution between risk and responsibility [128].

A possible limitation of the current study is that the search strategy used means that studies were included only if the authors explicitly linked the climate-related stressor they were considering and climate change. Indeed, a large amount of literature exists concerning the association between disasters and mental health [129] (without explicitly linking an increase in frequency and severity to climate change) which is likely to add value to the field. The geographical origin of the literature reviewed in the current paper is skewed towards high-income countries and unrepresentative of many regions of the world. The reasons for the relative abundance of research in countries such as Australia is worth exploring. Perhaps one reason for an under-representation of research from certain regions is that we only assessed studies in English. The decision to not explore non-English studies was related to our limited resources and capacity combined with the anticipation this extended search would yield limited studies.

It is also important to acknowledge that existing quality assessment tools are largely subjective and not perfectly adapted to the designs of studies included in this review and we were not able to assess the quality of studies employing modelling, case report, time-series, case-crossover, panel, or other unique study designs. Overall, our quality assessments highlight a need for improvement in future research—notably concerning improved measures of environmental exposures and mental health outcomes. For example, assessing the sensitivity and specificity of self-reported tools, for a given context and a given environmental hazard exposure, would be beneficial for assessing the reliability of such tools and allowing comparison across populations and studies.

The increasing number of studies on climate change and mental health parallels the growing traction in the broader field of climate change and health. Since 2007, the academic literature on this topic has tripled and media reports have increased by 78% [130]. However, mental health remains in a secondary position to physical health concerns, reflecting the state of global mental health more generally—despite research advances, the substantial burden of disease attributable to mental disorders has not improved, the quality of mental health services is routinely worse than services for physical health and government investment remains extremely limited [131]. This neglect is apparent in our findings and results in a continued lack of progress in improving the mental health of populations. The inattention to climate change impacts on mental health found in our scoping review echoes that noted by the Lancet Countdown report in 2018 [130]. This report noted that of the 16 national health adaptation strategies reviewed, mental health was the least considered climate-sensitive health outcome (mentioned by five out of 16 national health adaptation strategies).

## 5. Conclusions

The current scoping review has shown that climate change and mental health represents a rapidly growing area of research; however, it is underdeveloped and will need to accelerate and broaden in scope to respond with evidence-based mitigation and adaptation strategies. Most of the research has been devoted to assessing the mental health risks due to climate change, with less applied research investigating practical issues such as identifying the most effective interventions and policies to safeguard mental health in the face of climate change. Most of the research conducted to date is quantitative, cross-sectional, and conducted in high-income countries—therefore somewhat limiting the interpretability and the generalisability of the findings. Despite these limitations, the existing evidence overwhelmingly points to a negative association between climate change and mental health. Future research should aim at producing more robust and methodologically sound evidence on the link between mental health and climate change while also bearing in mind the importance of investigating what interventions, policies, and decision-making mechanisms can be put in place to mitigate the mental health impacts of climate change. This should represent a key priority in mental health research as, just like with physical health, climate change represents the biggest global threat to mental health of the 21st century [1].

## Appendix A

### Appendix A.1. Protecting Health from Climate Change—Global Research Priorities from the World Health Organization (WHO 2009)

(I) Assessing the risks
  - Improved evaluation of current climate-related health risks, rather than a primary focus on risks over very long timeframes
  - Identification of vulnerable populations and life stages
  - Quantification of the fraction of morbidity and mortality attributable to climate hazards, and to climate change
  - Better assessment of neglected climate-mental health linkages
(II) Identifying the most effective interventions
  - Systematic reviews of the evidence base for interventions
  - Methodological research to improve analytical tools for cost-effectiveness analysis
(III) Guiding health-promoting mitigation and adaption decisions in other sectors
  - Improved methods for assessment of the health implications of decisions in other sectors
  - Health implications of climate change mitigation: energy and transport sectors
  - Health implications of climate change adaptation: water, food and agriculture sectors
  - Improved integration of climate change mitigation, adaptation, and health through “settings-based” research
(IV) Improving decision-support
  - Research to improve vulnerability and adaptation assessments
  - Improvement of operational predictions
  - Improved understanding of decision-making processes
(V) Estimating the costs of protecting health from climate change
  - Definition of harmonized methods to estimate costs and benefits
  - Assessment of the health costs of inaction and the costs of adaptation
  - Improved economic assessment of the health co-benefits of climate change mitigation

### Appendix A.2. Search Strings by Online Database

**PsycINFO:** (((**IndexTermsFilt**: (“Atmospheric Conditions”) *OR* **IndexTermsFilt**: (“Climate Change”) *OR* **IndexTermsFilt**: (“Global Warming”)) *OR* (**title**: (“global warming”)) *OR* (**abstract**: (“global warming”)) *OR* (**title**: (“climate change”)) *OR* (**abstract**: (“climate change”)) *OR* (**title**: (“greenhouse effect”)) *OR* (**abstract**: (“greenhouse effect”)) *OR* (**title**: (weather)) *OR* (**abstract**: (weather))) *AND* ((**Year**: [2001 TO 2019]) *AND* **PublicationTypeFilt**: “Peer Reviewed Journal” *AND* ((**PopulationGroupFilt**: (“Human”))))) *AND* (((**IndexTermsFilt**: (“Mental Disorders”) *OR* **IndexTermsFilt**: (“Mental Health”)) *OR* (**title**: (“mental disorders”)) *OR* (**abstract**: (“mental disorders”)) *OR* (**title**: (“mental disorder”)) *OR* (**abstract**: (“mental disorder”)) *OR* (**title**: (“mental illness”)) *OR* (**abstract**: (“mental illness”)) *OR* (**title**: (“mental illnesses”)) *OR* (**abstract**: (“mental illnesses”)) *OR* (**title**: (“mental health”)) *OR* (**abstract**: (“mental health”))) *AND* ((**Year**: [2001 TO 2020]) *AND* **PublicationTypeFilt**: “Peer Reviewed Journal” *AND* ((**PopulationGroupFilt**: (“Human”)))))

**EMBASE:** (‘climate’/exp OR ‘greenhouse effect’/exp OR ‘weather’/exp OR ‘high temperature’/exp OR ‘climate change’:ab,ti OR ‘global warming’:ab,ti OR ‘greenhouse effect’:ab,ti OR ‘weather’:ab,ti) AND (‘mental disease’/exp OR ‘mental health’/exp OR ‘mental disorders’:ab,ti OR ‘mental disorder’:ab,ti OR ‘mental illness’:ab,ti OR ‘mental illnesses’:ab,ti OR ‘mental health’:ab,ti) AND [english]/lim AND [embase]/lim NOT [medline]/lim AND [2001–2020]/py

**Web of Science:** TS=(global warming OR climate change OR greenhouse effect OR weather) AND TS=(mental disorder OR mental disorders OR mental illness OR mental illnesses OR mental health) *AND* LANGUAGE: (English) *Indexes=SCI-EXPANDED, SSCI, A&HCI, CPCI-S, CPCI-SSH, BKCI-S, BKCI-SSH, ESCI, CCR-EXPANDED, IC Timespan=2001–2020*

**Scopus:** ((TITLE-ABS-KEY (“climate change”) OR TITLE-ABS-KEY (“global warming”) OR TITLE-ABS-KEY (“greenhouse effect”) OR TITLE-ABS-KEY (weather) AND (( TITLE-ABS-KEY (“mental disorder”) OR TITLE-ABS-KEY (“mental illness”) OR TITLE-ABS-KEY (“mental health”)) AND PUBYEAR > 2000 AND PUBYEAR < 2021)

**CINAHL:** (MH “Weather+”) OR (MH “Climate+”) OR TI(“climate change” or “global warming” or “greenhouse effect” or weather) OR AB(“climate change” or “global warming” or “greenhouse effect” or weather) AND (MM “Mental Health”) OR (MH “Behavioral and Mental Disorders+”) OR TI (“mental disorders” or “mental disorder” or “mental health” or “mental illness” OR “mental illnesses”) OR AB (“mental disorders” or “mental disorder” or “mental health” or “mental illness” OR “mental illnesses”) **Limiters** - Published Date: 20010101-20201231; English Language; Peer Reviewed; Exclude MEDLINE records

**Table A1:** Summary of original research (N = 120).

**Assessing the Risks (*n* = 101)**								
**Authors and Date**	**Country**	**Study Period**	**Study Design**	**Study Population**	**Sample Size**	**Outcomes**	**Exposure**	**Quality Rating**
Acharibasam and Anuga 2018	Ghana	NR	Cross-sectional	Farmers from three districts in Northern Ghana	180	Emotional regulation practices (given psychological distance i.e., geographical, social)	Self-reported exposure to excessive heat, declined rainfall, prolonged drought, excessive floods, and strong wind using questionnaires	Fair
Aguglia, Serafini et al., 2020	Italy	2013–2015	Cross-sectional	Psychiatric patients in Turin	730	Involuntary admissions	Interpolated exposure using daily meteorological data on wind, temperature, humidity, barometric pressure, solar radiation, rain, and hours of sunshine from Italian Meteorology’s Climate Data Service of Physics Department of the University of Turin	Good
Albrecht, Sartore et al., 2007	Australia	2003–2006	Cross-sectional	Residents of the Upper Hunter region of NSW and rural NSW	60	Solastalgia and environmental distress	Self-reported exposure to drought and mining using interviews, focus groups, and surveys	Fair
Allen 2020	USA	NR	Cross-sectional	Social workers in Alaska	159	Social workers’ attitudes about climate change and perceptions of the effects of climate change on their clients	Self-reported exposure to climate change using surveys	Poor
Anderson 2009	Australia	2004–2007	Phenomenological	Women health workers from Mallee, Victoria	2	How drought and climate change impact rural mental health, and how discourse of endurance and uncertainty can exacerbate problems in rural social work.	Self-reported exposure to drought using oral histories	Good
Asugeni, MacLaren et al., 2015	Solomon Islands	NR	Cross-sectional	Residents of six remote villages in East Malaita	57	Mental health	Self-reported exposure to sea-level rise using questionnaires	Good
Austin, Handley et al., 2018	Australia	2007–2013	Cohort	Farmers in NSW	664	Concerns about climate change	Self-reported exposure to climate change using surveys	Good
Austin, Rich et al., 2020	Australia	2007–2013	Cohort	Rural residents in NSW	823	Personal drought-related stress (PDS), community drought-related stress (CDS), and general psychological distress (K10)	Interpolated exposure using monthly meteorological data on rainfall to assess drought conditions	Good
Ayeb-Karlsson, Kniveton et al., 2020	Bangladesh	2014–2015	Mixed-method: Q-methodology and discourse analysis	Residents of Bhola Slum, Dhaka	62	Wellbeing and immobility	Self-reported exposure to climate-induced noneconomic losses using storytelling methodology	N/A
Ayeb-Karlsson 2020	Bangladesh	2014–2016	Case study	Residents of Bhola Slum, Dhaka	140	Immobility decision-making and wellbeing	Self-reported exposure to climate-induced immobility using Q-based discourse analysis	Fair
Ayeb-Karlsson 2020	Bangladesh	2014–2016	Case study	Residents of three coastal cyclone affected study sites of Bangladesh (Dalbanga South, Mazer Char and Gabtola).	280	Emotional fear and mental trauma	Self-reported exposure to climate-induced immobility using Q-based discourse analysis	Fair
Berry and Peel 2015	Australia	2013	Cross-sectional	Residents of regional and rural Australia (all states except Tasmania)	6674	Psychological distress (K10), emotional well-being (life satisfaction, happiness, and optimism), CC attitudes (belief, worry, distrust)	Self-reported exposure to climate change using surveys	Good
Billiot 2018	USA	NR	Cross-sectional	Indigenous community in South Louisiana	160	Health (physical, emotional, mental)	Self-reported exposure to environmental changes (including foul smelling air, noise, heavy vehicle movement, pollution, heritage destruction, soil erosion, loss of native fisheries) using surveys	Good
Bryan, Ward et al., 2020	United Kingdom	NR	Case study	Community members of seven different river catchments	41	Health and well-being	Self-reported exposure to drought using semi-structured and narrative interviews	Good
Bunce 2016	Canada	2015	Case study	Inuit women in Iqaluit, Nunavut	42	Vulnerability and adaptive capacity	Self-reported exposure to climate change using interviews and focus groups	Good
Burke, González et al., 2018	USA and Mexico	USA 1968–2004 Mexico1990–2010	Modelling	Populations of USA and Mexico	USA = 851,088; Mexico = 611,366	Suicide rates and depressive language	Interpolated exposure using gridded (4 × 4 km) daily meteorological data, from <10,000 weather stations, on temperature and precipitation from PRISM for the US; gridded daily (Berkeley Earth Surface Temperature Dataset) and monthly (Temperature and Precipitation: 1900–2010 Gridded Monthly Time Series Version 3.02, University of Delaware) temperature and precipitation data for Mexico	N/A
Carleton 2017	India	1967–2013	Modelling	Population of India	1472 deaths per 100,000	Suicide rates	Interpolated exposure using gridded (1 × 1 degree) daily meteorological data on temperature from the National Center for Environmental Prediction and gridded (0.5 × 0.5 degree) monthly cumulative precipitation data from the University of Delaware	N/A
Carlsen, Oudin et al., 2019	Sweden	2012–2017	Case-crossover	Residents of Gothenburg	84,230	Daily psychiatric emergency visits	Interpolated exposure using daily meteorological data, from an urban background measuring station, on temperature from the Swedish Meteorological and Hydrological Institute data repository	N/A
Carnie, Berry et al., 2011	Australia	2008–2009	Case study	Young people, teachers, and service providers in rural drought-affected NSW	96	Mental health	Self-reported exposure to drought using consultative forums	Good
Chan, Lam et al., 2018	Hong Kong	2002–2011	Ecological	Public hospital admissions	N/A	Mental-disorder hospitalisation	Interpolated exposure using daily meteorological data, from a monitoring station near the centre of Hong Kong, on temperature and humidity from the Hong Kong Observatory and daily average level of air pollutants, from all general air quality monitoring stations except one rural location, from the Environmental Protection Department of Hong Kong	Good
Chen, Lin et al., 2019	Taiwan	2003–2013	Cohort	Taiwan health insurance beneficiaries	945,171	MDD diagnosis	Interpolated exposure using daily meteorological data, from the weather monitoring station nearest the residential township of subjects, on temperature, sunshine duration, and precipitation from the Taiwan Central Weather Bureau	Good
Cooper, Hutchings et al., 2019	Ethiopia	2017–2018	Cross-sectional	Members of three villages in Afar region: Eger, Tirtira, Adkonta	36	Emotional wellbeing	Self-reported exposure to water insecurity using focus groups and interviews	Good
Cunsolo Willox, Harper et al., 2012	Canada	2009–2010	Case study	Residents of the Inuit community of Rigolet, Nunatsiavut	112	Lived experiences of ecological grief and loss	Self-reported exposure to caribou decline using interviews	Good
Cunsolo Willox, Harper et al., 2013	Canada	2010	Case study	Residents of the Inuit community of Rigolet, Nunatsiavut, community-based health workers and Nunatsiavut Government health professionals	67	Sense of place, health, and well-being	Self-reported exposure to climate change using interviews	Good
Cunsolo, Borish et al., 2020	Canada	NR	Case study	Inuit from the Nunatsiavut and NunatuKavut regions	105	Mental health and well-being	Self-reported exposure to climate change using interviews	Good
Dean and Stain 2010	Australia	2007	Cross-sectional	Adolescents (aged 11–17) from five schools in rural NSW	111	Emotional well-being (Strengths and Difficulties Questionnaire) and perceived impacts of drought (Drought and Community Survey for Children)	Self-reported exposure to drought using focus groups and surveys	Fair
Di Giorgi, Michielin et al., 2020	Italy	NR	Cross-sectional	Migrants from African countries with extreme and high vulnerability to climate change in Northern Italy	100	Perception of climate change, loss of social capital, and mental health (depressive and anxiety symptoms)	Self-reported exposure to climate change using interviews	Fair
Di Nicola, Mazza et al., 2020	Italy	2016–2019	Cross-sectional	Euthymic bipolar patients in Rome	704 (352 bipolar subjects, 352 healthy controls)	Weather sensitivity (assessed using METEO-Questionnaire) and its relationship to suicide attempts	Self-reported exposure to weather and climate variation using questionnaires	Good
Ding, Berry et al., 2016	Australia	2006–2008	Cross-sectional	Residents of NSW aged 45+	53,144	Psychological distress (K10) and having been treated for depression or anxiety	Interpolated exposure using gridded (5 × 5 km) daily meteorological data, based on in situ observations and topography-resolving analysis, on temperature and vapour pressure (to approximate humidity) from the Australian Bureau of Meteorology	Good
Dodd, Scott et al., 2018	Canada	2015	Phenomenological	Residents of four Northwest Territories communities	30	Mental and emotional well-being, physical health, and livelihoods	Self-reported exposure to wildfires using interviews	Good
du Bray 2018	USA, Fiji, Cyprus, New Zealand, England	2014	Ethnography	Biophysically vulnerable communities in the US (Mobile, Alabama; Kodiak, Alaska; Phoenix, Arizona), Fiji (Viti Levu), Cyprus (Nicosia), New Zealand (Wellington), England (London	Total: 375 (USA: 103 Fiji: 68 Cyprus: 40 New Zealand: 86 London: 78)	Emotional impacts	Self-reported exposure to climate change using interviews and surveys	Good
Durkalec, Furgal et al., 2015	Canada	2010–2011	Case study	Residents of the Inuit community of Nain, Nunatsiavut	22	Health	Self-reported exposure to sea ice using focus groups and interviews	Good
Eisenman, McCaffrey et al., 2015	USA	2012	Cross-sectional	Residents of five communities affected by the Wallow Fire in Arizona	416	Psychological distress (K10) and solastalgia	Self-reported exposure to wildfires using surveys	Good
Ellis and Albrecht 2017	Australia	2013–2014	Case study	Family farmers in Newdegate, WA	22	Place-related mental health and wellbeing	Self-reported exposure to climate change using interviews	Good
Farrokhi, Khankeh et al., 2020	Iran	2018–2019	Case study	Not specified	33	Self-reported emotional and mood effects	Self-reported climate change risk perception using interviews	Fair
Fearnley, Magalhaes et al., 2014	Australia	2006–2008	Cross-sectional	Women in south-west WA	879	Mental health component scores	Interpolated exposure using environmental data, based on proportional mapping, on dryland salinity from the Western Australian Department of Agriculture’s soil and landscape mapping database, and gridded (5 × 5 km) land surface temperature and normalised difference vegetation index (proxy for rainfall) from the National Oceanographic and Atmospheric Administration’s (NOAA) Advanced Very High Radiometer	Good
Fischer and Van de Vliert 2011	Global	N/A	Modelling	Nonclinical adult populations	N/A	Indicators of general health complaints, burnout, state and trait anxiety, and depression	Interpolated exposure; monthly temperature deviations calculated at the country level but data source not provided.	N/A
Flint, Robinson et al., 2011	USA	2008–2010	Participatory research	Residents of three remote Alaska Native communities	Interviews: 65 Surveys: 116	Perceived health status and impacts of berries, measured health benefits of berries, perceived environmental and community wellness	Self-reported exposure to environmental changes using interviews, focus groups, and surveys	Good
Friel, Berry et al., 2014	Australia	2007–2008	Cross-sectional	Nationally representative sample of Australian residents aged 15 years and over	5012	Psychological distress (K10) and three indicative measures of food insecurity	Interpolated exposure using gridded (0.25 degree) monthly meteorological data on rainfall (used to calculate Hutchinson Drought Indices) from the Australian Bureau of Meteorology	Good
Fuentes, Asselin et al., 2020	Canada	2016	Cross-sectional	Members of four Indigenous communities in the eastern Canadian boreal forest	251	Environmental distress (using questionnaire based on the Environmental Distress Scale (EDS) and the Connor–Davidson Resilience Scale (CD-RISC-10))	Self-reported exposure to environmental changes using questionnaires	Good
Gibson, Haslam et al., 2019	Tuvalu	2015	Case study	Residents of Tuvalu and key informants	39	Psychological distress and associated impairment	Self-reported exposure to climate change using interviews	Good
Gibson, Barnett et al., 2020	Tuvalu	2016	Cross-sectional	Tuvaluan residents in Funafuti	100	Determinants and idioms of distress and culturally prescribed responses to coping with distress	Self-reported exposure to climate change using interviews	Fair
Gu, Zhang et al., 2020	China	2009–2018	Modelling	Residents in Ningo, China	372,027	Mortality	Interpolated exposure using daily meteorological data, from Yinzhou station (Lat. 29.79 N, Long. 121.55 E; the only national basic weather monitoring station in the central urban area of Ningbo) on temperature and humidity from the Ningbo Meteorological Bureau	N/A
Hanigan, Schirmer et al., 2018	Australia	2015	Cross-sectional	Adults living in rural areas in Victoria	5312	Psychological distress (K10)	Interpolated exposure using monthly meteorological data on rainfall (used to calculate Hutchinson Drought Indices) from the Australian Bureau of Meteorology	Good
Hansen, Bi et al., 2008	Australia	1993–2006	Ecological	Residents of Adelaide metropolitan area	171,614	Hospital admissions and mortality attributed to mental, behavioural, and cognitive disorders	Interpolated exposure using daily meteorological data, from a central city weather station, on temperature from the Australian Bureau of Meteorology	Good
Helama, Holopainen et al., 2013	Finland	1751–2008	Modelling	Finnish population	94,356	Suicide	Interpolated exposure using meteorological data on temperature from the Uppsala temperature record [Department of Meteorology, Uppsala University]	N/A
Helm, Pollitt et al., 2018	USA	NR	Cross-sectional	Adults who have at least one child	342	Depressive symptoms and pro-environmental behaviours	Self-reported exposure to environmental concern using surveys	Fair
Hoffmann, Oliveira et al., 2016	Brazil	2014	Case report	Individual patient with schizophrenia on clozapine treatment	1	Heat stroke	Not applicable	N/A
Howard, Ahmed et al., 2020	USA	2017	Cross-sectional	Farmers and ranchers in Montana	125	Climate risk perception and mental well-being, including climate-related anxiety	Self-reported exposure to climate change using surveys	Fair
Jones, Wootton et al., 2012	Australia	2008–2009	Cross-sectional	Patients with OCD checking subtype in Sydney	50	OCD symptoms	Self-reported exposure to climate change using clinical interviews	Fair
Kabir 2018	Bangladesh	2015–2016	Case study	Residents and health professionals in the Hill-Tracts region	125	Psychological health and well-being	Self-reported exposure to climate change using interviews	Fair
Khalaj, Lloyd et al., 2010	Australia	1998–2006	Case-only	Residents of Sydney East and West, Illawarra, Gosford-Wyong, and Newcastle	1,497,655	Emergency hospital admissions and underlying conditions	Interpolated exposure using daily meteorological data, from a monitoring station in each of the five regions of interest, on temperature (used to examine extreme heat) from the NSW Bureau of Meteorology	N/A
Kim, Lim et al., 2015	South Korea	1992–2009	Modelling	Residents of Seoul	271,633	Mortality (relative risks and attributable deaths)	Interpolated exposure using daily meteorological data, from the representative synoptic surface observation station in Seoul, on humidity, air pressure, and temperature from the Korea Meteorological Administration	N/A
Li, Ferreira et al., 2020	USA	1993–2010	Cross-sectional	USA adult population	3,060,158	Self-reported mental health	Interpolated exposure using gridded (4 × 4 km) daily meteorological data on temperature and precipitation from PRISM	Good
Lindvall, Kinsman et al., 2020	Somalia, Kenya and Ethiopia	2018	Case study	Stakeholders from different disciplines (climate, weather, environment, migration, and public health) and agencies (national, regional, and local; governmental, non-governmental, and UN)	39	Health and health care access	Self-reported exposure to drought-related migration using interviews	Good
Liu, Liu et al., 2018	China	2010	Case-crossover	Residents of Jinan	19,569	Daily hospital visits for mental illness	Interpolated exposure using daily meteorological data on temperature (used to define heatwave events), humidity, wind, and air pressure from the China Meteorological Data Sharing Service System	N/A
Mason, Erwin et al., 2018	USA	2016	Cross-sectional	Primarily low- and moderate-income residents of Knoxville, Tennessee	424	Self-reported mental health and potential mediating factors including feeling prepared for extreme weather, general health, income, social cohesion, and concern about climate change	Self-reported exposure to summer heat waves and extreme winter weather using surveys	Fair
Mason, Sharma et al., 2020	USA	2016	Cross-sectional	Low- to middle-income adults of different racial groups in Knoxville, Tennessee	426	Physical and mental health, associations with human, financial, physical, and social capital	Self-reported exposure to summer heat waves and extreme winter weather using surveys	Fair
Mayner, Arbon et al., 2010	Australia	2009	Cross-sectional	Emergency department patients in Adelaide	9244	ED patient presentations	Interpolated exposure using meteorological data in Adelaide was used to define two types of heat waves events (Australian Bureau of Meteorology criterion)	Good
McNamara and Westoby 2011	Australia	2009–2010	Case study	Aunties (respected older women) living on Erub Island, Torres Strait	4	Emotional, cultural, psycho-social impacts	Self-reported exposure to climate change using interviews	Good
Middleton, Cunsolo et al., 2020	Canada	2012–2013	Case study	Inuit community members and health professionals in Nunatsiavut	116	Mental health and wellness	Self-reported exposure to changing weather and seasonal patterns using interviews	Good
Mulchandani, Armstrong et al., 2020	England	2015–2017	Cohort	People living in neighbourhoods affected by flooding in the south of England between 1 December 2013, and 31 March 2014	819	Depression, anxiety, and PTSD	Self-reported exposure to flooding using questionnaires	Good
Ng, Wilson et al., 2015	Australia	NR	Case study	Adults living in four rural communities in NSW	46	Wellbeing	Self-reported exposure to flood and drought using interviews and focus groups	Good
Nitschke, Tucker et al., 2007	Australia	1993–2006	Case-series	Residents of Adelaide metropolitan area	N/A	Ambulance transports, hospital admissions, and mortality	Interpolated exposure using daily meteorological data, from Kent Town station, on temperature (used to define heatwave events) from the Australian Bureau of Meteorology	Good
Niu, Gao et al., 2020	China	2016–2018	Time-series	Patients with mental disorders residing in Beijing	16,606	Daily emergency visits related to mental disorders	Interpolated exposure using daily meteorological data, from three fixed-site stations in Beijing, on temperature, humidity, duration of sunshine, barometric pressure, precipitation, and wind from the China Meteorological Data Service Center	N/A
Noelke, McGovern et al., 2016	USA	2008–2013	Cross-sectional	Population aged 18+	1,900,000	Emotional well-being	Interpolated exposure using gridded (0.125 × 0.125 degree) daily meteorological data on temperature from the North American Land Data Assimilation System	Good
Obradovich, Migliorini et al., 2018	USA	2002–2012	Cross-sectional	USA residents	1,961,743	Mental health difficulties	Interpolated exposure using gridded (4 × 4 km) daily meteorological data on temperature and precipitation from PRISM, and average daily data on cloud cover, humidity, and wind from the National Centers for Environmental Prediction Reanalysis II project	Good
OBrien, Berry et al., 2014	Australia	2007–2008	Cross-sectional	Nationally representative sample of Australian residents aged 15 years and over	5012	Psychological distress (K10)	Interpolated exposure using daily meteorological data on rainfall (used to calculate Hutchinson Drought Indices) from the Australian Bureau of Meteorology	Good
Ogunbode, Böhm et al., 2019	United Kingdom	2013–2014	Cross-sectional	British sample (this study only used a sub-sample of a larger questionnaire. They only used individuals who indicated that they had been personally affected a little, a fair amount, or a great deal by the 2013/2014 UK winter flooding	821	Negative emotions and mitigation intention	Self-reported exposure to flooding using surveys	Good
Oudin Astrom, Schifano et al., 2015	Italy and Sweden	2000–2008	Cohort	Residents of Rome and Stockholm aged 50 years or older	110,531	Mortality	Interpolated exposure using daily meteorological data, from the airport station closest to the city in Rome and the station at Bromma City aiport in Stockholm, on temperature (used to define heatwave events)	Good
Page, Hajat et al., 2012	England	1998–2007	Cohort	Patients with primary diagnoses of psychosis, dementia, alcohol misuse, and other substance misuse	22,562	Mortality	Interpolated exposure using daily meteorological data, from all monitoring stations in England, on temperature from the British Atmospheric Data Centre	Good
Pailler and Tsaneva 2018	India	2003 & 2007	Cross-sectional	Sample of 8468 from World Health Survey (WHS) in 2003 and 7759 from 2007 Study on global AGEing and adult health (SAGE)	16,227	Depression; stress (cognitive and sleep difficulties, ability to cope); agency (control)	Interpolated exposure using gridded (0.5 × 0.5 degree) monthly meteorological data on temperature and precipitation (used to define weather extremes) from the Matsurra & Willmott ‘Terrestrial air temperature and precipitation: Monthly and annual time series (1950–1999)’	Good
Polain, Berry et al., 2011	Australia	2008	Case study	Older farmers in NSW	152	Mental health	Self-reported exposure to drought using consultative forums	Good
Powers, Dobson et al., 2015	Australia	1996–2008	Cohort	Women living in rural Australia	6664	Mental Health Index scores (from the Medical Outcomes Study Short Form 36-SF36)	Interpolated exposure; the Hutchinson Drought Index was used but data source not provided.	Good
Powers, Loxton et al., 2012	Australia	2004	Cross-sectional	Women living outside major cities	6584	SF-36 General Health (GH) and Mental Health (MH) scores, and perceived stress	Interpolated exposure to Exceptional Circumstances declared area using the 2004 survey with latitude and longitude declared areas in the same year for each participant.	Good
Preti, Lentini et al., 2007	Italy	1974–2003	Modelling	Italians	97,693	Suicide	Interpolated exposure using monthly meteorological data on temperature anomalies from the Istituto di Scienze dell’Atmosfera e del Clima (Institute of Atmospheric Sciences and Climate)	N/A
Proverbs, Lantz et al., 2020	Canada	2017	Case study	Members of four Gwich’in First Nation communities in the Northwest Territories	29	Well-being	Self-reported exposure to access to fish using interviews	Good
Qi, Hu et al., 2014	Australia	1986–2005	Modelling	Population of Australia	45,293	Suicide rates	Interpolated exposure using monthly meteorological data, from monitoring stations in each Local Government Area, on rainfall, humidity, and temperature from the Australian Bureau of Meteorology	N/A
Rahman, Mohamad et al., 2014	Malaysia	2012	Cross-sectional	University students in Malaysia	200	Perceived awareness and perceived physical and psychological impact	Self-reported exposure to climate change using surveys	Good
Rigby, Rosen et al., 2011	Australia	2008	Case study	Aboriginal communities in NSW	166	Social and emotional well-being, and possible adaptive strategies	Self-reported exposure to drought using consultative forums	Good
Rotge, Fossati et al., 2014	Global	2001–2005	Ecological	Populations of 17 countries: Belgium, Colombia, France, Germany, Israel, Italy, Japan, Lebanon, Mexico, Netherlands, New Zealand, Nigeria, PR China, South Africa, Spain, Ukraine, United States	85,052	Lifetime prevalence of mood disorders	Interpolated exposure using monthly meteorological data on temperature and rainfall from the World Bank Group	Fair
Sartore, Kelly et al., 2008	Australia	NR	Case study	Two rural communities in NSW	39	Emotional and social wellbeing	Self-reported exposure to drought using focus groups	Good
Searle and Gow 2010	Australia	2008	Cross-sectional	University students and members of the general public in Queensland	275	Climate change distress; environmental beliefs (NEP); depression, anxiety, and stress (DASS-21); future anxiety (FAS3); intolerance of uncertainty (IUS-12); religiosity (SCSRFQ-SF)	Self-reported exposure to climate change using questionnaires	Good
Sim, Kim et al., 2020	Japan	1972–2015	Time-series	Population of Japan	1,067,333	Suicide	Interpolated exposure using daily meteorological data, from a weather station in the capital city of each prefecture, on temperature and humidity from the Japan Meteorology Agency	N/A
Stain, Kelly et al., 2011	Australia	2006–2009	Cross-sectional	Adults living in rural NSW	302	Psychological distress (K10) and an index of concern or worry about drought	Interpolated exposure using gridded (0.25 degree) monthly meteorological data on rainfall (used to calculate drought exposure) from the Australian Bureau of Meteorology	Good
Sugg, Dixon et al., 2019	USA	2013–2017	Time-series	Adolescents and young adults in Atlanta, Georgia; Chicago, Illinois; Los Angeles, California, and New York City; New York.	59,000	Crisis support-seeking behaviour	Interpolated exposure using daily meteorological data, from the following international airport weather stations: Atlanta Hartsfield, Chicago O’Hare, LaGuardia, and Los Angeles, on temperature from the Applied Climate Information System available from and maintained by the National Oceanic and Atmospheric Administration’s Regional Climate Centers	N/A
Tasdik Hasan, Adhikary et al., 2020	Bangladesh	2015	Case study	Key informants and cyclone-affected people in Koyra Sadar Upazila, Khulna district	20	Perceived need for mental health support and availability of such services	Self-reported exposure to cyclones using interviews	Good
Tawatsupa, Lim et al., 2010	Thailand	2005	Cross-sectional	Workers	40,913	Psychological distress (K10) and overall health (first question of SF8)	Self-reported exposure to heat stress using questionnaires	Fair
Trang, Rocklov et al., 2016	Vietnam	2008–2012	Time-series	Patients with mental disorders in the north of Vietnam	21,443	Hospital admissions for mental disorders	Interpolated exposure using daily meteorological data, from several monitoring stations in Hanoi, on temperature (used to define heatwave events)	N/A
Trinh, Feeny et al., 2020	Vietnam	2002–2013	Modelling	Children	2000	Stunting and underweight in children, and parental mental health as a mediating factor	Interpolated exposure using gridded (0.5 × 0.5 degree) monthly meteorological data on rainfall from the National Oceanic and Atmospheric Administration	N/A
Verplanken and Roy 2013	Europe and USA	2012	Participatory research	Participants who took survey in USA and Europe	132	Habitual ecological worrying	Self-reported exposure to climate change using surveys	Fair
Vida, Durocher et al., 2012	Canada	1995–2007	Modelling	Patients aged 15 years and older in Quebec	347,552	Emergency department visits for mental and psychosocial problems	Interpolated exposure using daily meteorological data, from available weather stations, on temperature and humidity from Environment Canada and the Ministry of Sustainable Development, Environment, and Parks	N/A
Wang, Lavigne et al., 2014	Canada	2002–2010	Ecological	Residents of Toronto	271,746	Emergency room (ER) visits related to mental and behavioural diseases (MBD)	Interpolated exposure using hourly meteorological data, from the monitoring station at Toronto Pearson International Airport, on temperature and humidity from Environment Canada	Good
Wei, Zhang et al., 2020	China	2005–2014	Ecological	Patients of Anhui Mental Health Center in Hefei	36,607	Admissions for schizophrenia	Interpolated exposure to precipitation data for the region Hefei and flood events were defined based on established criteria.	Poor
Williams, Hill et al., 2016	New Zealand	1993–2009	Modelling	Population of New Zealand	47,265	Self-harm hospitalisations	Interpolated exposure using gridded (5 km) daily meteorological data, from a virtual climate network, on temperature from the National Institute of Water and Atmospheric Research	N/A
Williams, Nitschke et al., 2012	Australia	1993–2009	Ecological	Population of Adelaide	N/A	Mortality, ambulance call-outs, emergency department (ED) presentations and hospital admissions	Interpolated exposure using daily meteorological data, from Kent Town station, on temperature from the Australian Bureau of Meteorology	Good
Xu, Wheeler et al., 2018	Australia	2008–2014	Cross-sectional	Australian children	6857	Mental health	Interpolated exposure using gridded daily meteorological data on temperature from the Australian Bureau of Meteorology	Good
Xu, Zhao et al., 2020	Brazil	2000–2015	Case-crossover	Brazilian population	49,145,997	All-cause and cause-specific hospitalisations	Interpolated exposure using gridded (0.25 degree) daily meteorological data on temperature, from a Brazilian meteorological dataset, with the analyses restricted to the hottest four consecutive months (hot season) for each city each year during the study period	N/A
Yazd, Wheeler et al., 2020	Australia	2001–2015	Panel	Farmers in the Murray-Darling Basin	235	Mental health, assessed using the mental health inventory (MHI-5), a sub-scale of the SF-36	Interpolated exposure using gridded monthly meteorological data on rainfall (used to define drought) from the Australian Bureau of Meteorology	N/A
Yoon, Oh et al., 2014	South Korea		2008, 2100	Modelling (burden of disease)	Population of South Korea	N/A	Burden of disease (DALYs) attributable to climate factors	Interpolated exposure using daily meteorological data, from 77 regional meteorological offices and observatories, on temperature from the Korea Meteorological Administration; ozone concentration data from the 2008 Annual Report of Ambient Air Quality in Korea; and the number of natural disasters was based on the Annual Report of Disasters in Korea	N/A
Zhao, Zhang et al., 2016	China	2005–2014	Ecological	Population of Hubei	36,607	Emergency hospital admissions for schizophrenia	Interpolated exposure using daily meteorological data on temperature and humidity from the Hefei Bureau of Meteorology	Good
**Identifying the Most Effective Interventions (*n* = 8)**								
**Authors and Date**	**Country**	**Study Period**	**Study Design**	**Study Population**	**Sample Size**	**Outcomes**		**Quality Rating**
Hart, Berry et al., 2011	Australia	2007–2010	Program development	All rural communities facing prolonged drought in NSW	3000	A community development model effective in helping communities build capacity and resilience in the face of chronic drought-related hardship.		N/A
Hayes, Poland et al., 2020	Canada	2018	Case study	Key informant health and social services leaders, front-line health and social services workers and community members who experienced the 2013 Southern Alberta flood	46	Health and social service responses to the long-term mental health impacts of the flood		Good
Heaney and Winter 2016	Tanzania	2013	Case study	Two demographically matched adult Maasai populations in Arusha: rural-to-urban migrants and permanent rural residents (i.e., non-migrants)	14	Health perceptions and help-seeking behaviours		Good
Netuveli and Watts 2020	United Kingdom	2009–2010	Panel	UK households	22,427 people in 9344 households	Life satisfaction, self-rated health, mental health (assessed using 12-item version of the General Health Questionnaire (GHQ-12)), physical quality of life and mental quality of life		N/A
Patrick and Capetola 2011	Australia	2010–2011	Case study	Victorian health care agencies that explicitly identified climate change as a priority	10	Health promotion practice		Good
Purcell and McGirr 2018	Australia	NR	Cross-sectional	Health service managers working in rural remote metropolitan areas in NSW	43	Perceptions and recommendations of rural HSMs on climate change and its impact on health in their local communities		Fair
Valois, Blouin et al., 2016	Canada	NR	Cross-sectional	Family physicians in Quebec	24	Family physicians’ educational needs regarding the health impacts of climate change		Good
Wang, Jiang et al., 2019	China	2018	Cross-sectional	Chinese residents	1273	Environmental complaint behaviour		Fair
**Guiding Health-Promoting Mitigation and Adaption Decisions in Other Sectors (*n* = 7**)								
**Authors and Date**	**Country**	**Study Period**	**Study Design**	**Study Population**	**Sample Size**	**Outcomes**		**Quality Rating**
Harper, Edge et al., 2015	Canada	2010	Participatory research	Inuit community members and government employees in the Nunatsiavut region	209	Climate-sensitive health issues		Good
MacFarlane, Shakya et al., 2015	Nepal	2014	Participatory research	Female subsistence farmers aged 27–49 years in the Jumla district	10	Mental health		Good
Petrasek MacDonald, Cunsolo Willox et al., 2015	Canada	2012–2013	Case study	Inuit youth in Nunatsiavut	17	Youth-specific protective factors that enhance mental health and well-being		Good
Raker, Arcaya et al., 2020	USA	2003–2018	Cross-sectional	Low-income primarily Black parents in New Orleans and Houston	113	Lessons learned from the RISK project, including planning priorities to mitigate the health consequences of disasters		Good
Sawatzky, Cunsolo et al., 2020	Canada	2015–2016	Case study	Inuit community members, government representatives and health professionals in Rigolet	31	Conceptual framework for an Inuit-led integrated environment and health monitoring system		Good
Sorgho, Mank et al., 2020	Burkina Faso	2018	Case study	Subsistence farmers	32	Perceptions of climate change and health; agricultural adaptation strategies including barriers and possibilities; dietary adaptation practices; sources of support (social and governmental)		Good
Wong-Parodi 2020	USA	2019	Cross-sectional	Individuals living in one of 41 California counties in PG&E’s and SCE’s (utility companies) public safety power shutoffs (PSPS) zone	328	Physical and mental health; views on the PSPS decisions; response to those decisions; views on wildfire risk		Fair
**Improving Decision-Support (*n* = 3)**								
**Authors and Date**	**Country**	**Study Period**	**Study Design**	**Study Population**	**Sample Size**	**Outcomes**		**Quality Rating**
McIver, Kim et al., 2016	Cook Islands, Federated States of Micronesia, Fiji, Kiribati, Marshall Islands, Nauru, Niue, Palau, Samoa, Solomon Islands, Tonga, Tuvalu, and Vanuatu	2010–2012	Vulnerability assessment	Pacific island countries	13 Pacific island countries	Health risks and adaptation strategies		N/A
Seidel and Bell 2014	Australia, Belgium, Wales, Finland, Denmark, Germany, United Kingdom, Russia, USA, Spain, Scotland, Netherlands	2012	‘Critical computational linguistics’	High-income countries	Exhaustive sample of national adaptation policy documents from Annex 1 (‘developed’) countries of the UNFCC	Representation of climate-vulnerable groups in adaptation policy		N/A
Valois, Caron et al., 2019	Canada	NR	Cross-sectional	Adults living in or near a designated flood-prone area in Quebec	1951	Behavioural indices of flood adaptation		Fair
**Estimating the Costs of Protecting Health from Climate Change (*n* = 1)**								
**Authors and Date**	**Country**	**Study Period**	**Study Design**	**Study Population**	**Sample Size**	**Outcomes**		**Quality Rating**
Maughan, Patel et al., 2016	United Kingdom	2011–2014	Retrospective observational study	Patients with a common mental health condition attending the Connect service; control group of patients attending same primary care service not attending Connect	Connect group = 30, control group = 29	Financial and environmental impacts of GP appointments, psychotropic medications and secondary-care referrals		Good

Abbreviations—NR: not reported; NSW: New South Wales; USA: United States of America; WA: Western Australia.
